# A duplex real-time RT-PCR assay for detecting H5N1 avian influenza virus and pandemic H1N1 influenza virus

**DOI:** 10.1186/1743-422X-7-113

**Published:** 2010-06-02

**Authors:** Xiao-ping Kang, Tao Jiang, Yong-qiang Li, Fang Lin, Hong Liu, Guo-hui Chang, Qing-yu Zhu, E-de Qin, Cheng-feng Qin, Yin-hui Yang

**Affiliations:** 1 State Key Laboratory of Pathogen and Biosecurity, Beijing Institute of Microbiology and Epidemiology, Beijing 100071, China

## Abstract

A duplex real-time reverse transcriptase polymerase chain reaction (RT-PCR) assay was improved for simultaneous detection of highly pathogenic H5N1 avian influenza virus and pandemic H1N1 (2009) influenza virus, which is suitable for early diagnosis of influenza-like patients and for epidemiological surveillance. The sensitivity of this duplex real-time RT-PCR assay was 0.02 TCID_50 _(50% tissue culture infective dose) for H5N1 and 0.2 TCID_50 _for the pandemic H1N1, which was the same as that of each single-target RT-PCR for pandemic H1N1 and even more sensitive for H5N1 with the same primers and probes. No cross reactivity of detecting other subtype influenza viruses or respiratory tract viruses was observed. Two hundred and thirty-six clinical specimens were tested by comparing with single real-time RT-PCR and result from the duplex assay was 100% consistent with the results of single real-time RT-PCR and sequence analysis.

## Findings

In March and April 2009, a novel swine-origin pandemic H1N1 influenza virus appeared and spread worldwide. The pandemic of H1N1 2009 influenza virus poses a public health threat. According to the World Health Organization (WHO), as of 30 April 2010, worldwide at least 17919 death cases have been reported [1]. Compared with influenza A H1N1 virus, the highly pathogenic avian influenza virus H5N1 has a much higher mortality rate: H5N1 has a mortality rate of more than 50% while novel pandemic H1N1 has only about 1% [2]. Nevertheless, pandemic H1N1 and highly pathogenic H5N1 manifest similar clinical symptoms at the early stage of infection [3]. Early detection of these two pathogens is an essential prerequisite for effective control and prevention of the pandemic.

Real-time RT-PCR/PCR is a powerful method for the sensitive and specific detection of virus-derived nucleic acids in clinical samples [4]. It is time-saving and more specific compared with endpoint PCR. Therefore, real-time RT-PCR assay has been widely used and recommended by WHO for many rapid viral pathogen detection. Moreover, multiplex assays allow to measure several fluorophores in one well, which can simultaneously detect different target sequences [5]. Since the outbreak of pandemic H1N1 influenza, many real-time RT-PCR assays have been developed for detecting of the novel H1N1 [6-13]. Some multiplex real-time RT-PCR assays for simultaneous typing (A/B) and subtyping of H1, H2, H3, H5, H7 and H9 of influenza A viruses have also been reported [14-17]. However, no studies were reported for subtyping of the novel pandemic H1N1 and H5N1 simultaneously.

In this study, a duplex TaqMan real-time RT-PCR assay was improved by adjusting the concentrations of primers and probes in the WHO protocols. The assay could simultaneously detect H5N1 avian influenza virus and pandemic H1N1 influenza virus, which could be used for early diagnosis of influenza-like patients and for epidemiological surveillance.

The primers and probes for the novel pandemic H1N1 and the influenza H5N1 were derived from the protocols recommended by WHO [18,19]. The sequence of primers for H5N1 were H5-sense: 5'-GGA ACT TAC CAA ATA CTG TCA ATT TAT TCA-3', H5-antisense: 5'-CCA TAA AGA TAG ACC AGC TAC CAT GA-3' and H5-probe: 5'-HEX-TTG CCA GTG CTA GGG AAC TCG CCA C-BHQ1-3' which was labeled with the reporter hexachloro-6-carboxyfluorescein (HEX) at the 5' end and a black hole quencher (BHQ1) at the 3' end [19]. The sequence of primers and probe for pandemic H1N1 were H1-sense: 5'-GTG CTA TAA ACA CCA GCC TYC CA-3', H1-antisense: 5'-CGG GAT ATT CCT TAA TCC TGT RGC-3', H1-probe: 5'-FAM-CAG AAT ATA CAT* CCR GTC ACA ATT GGA RAA-3' which was labeled with the reporter 6-carboxyfluorescein (6-FAM) at the 5' end and a black hole quencher (BHQ1) at the a modified T residue [18]. All primers and probes were synthesized by Dalian Takara Company.

RNA was extracted from the supernatants of cultured viruses or from clinical specimens by RNeasy Mini kit (Qiagen, Hilden, Germany) according to the manufacture's instruction. Virus RNA extractions were conducted in Biosafety Level 3 (BSL-3) facilities.

The duplex real-time RT-PCR amplification was carried out in a 20 μL volume reaction with the Quantitect Probe RT-PCR kit (Qiagen, Hilden, Germany) with the LightCycler 2.0 system (Roche, Mannheim, Germany). Different concentrations of the primers and probes for both H5N1 and H1N1 were combined and adjusted to improve the sensitivity of the assay [20]. The concentrations of primers and probe for H5 gene in the duplex system was optimized by using a serial of 10-fold diluted H5N1 RNAs as templates. The sensitivity of the duplex assays was evaluated at the concentration of H5 primers as 0.4 μM, 0.8 μM, 1.6 μM and 3.2 μM. The results showed that the sensitivity of the duplex assays dramatically increased with the concentration of H5 primers. The duplex assay had the best detecting result at the H5 primer concentration of 1.6 μM. The concentration of probes for H5 gene was also optimized by examining the concentration at 0.1 μM, 0.2 μM, 0.4 μM and 0.8 μM, and any significant different results at different concentrations was not observed. Therefore, 0.1 μM was selected as the final concentration of H5 probe in the duplex real-time RT-PCR system. Similar to the duplex assay for H5 gene, the concentrations of primers and probe for H1 also were optimized by using RNA from 2, 0.2, 0.02 and 0.002 TCID_50 _H1N1 as templates. The concentration of H1 primers was optimized from 0.1 μM, 0.2 μM, 0.4 μM, 0.8 μM and 1.6 μM, while the concentration of H1 probe was optimized at 0.1 μM, 0.2 μM, 0.4 μM and 0.8 μM. Thus, 0.4 μM of primers and 0.2 μM of probe were selected as the optimal concentration for H1 gene. The reactions were incubated at 50°C for 30 min, followed by 95°C for 10 min, 45 cycles of 95°C for 15 s, and 52°C for 1 min. Fluorescence was recorded at 52°C.

The final optimized reaction mixture consisted of 10 μL of 2× reaction buffer, 0.2 μL reverse transcription enzyme, 1.6 μM of each H5N1 primer, 400 nM of each novel H1N1 primer, 100 nM of H5N1 probe, 200 nM of pandemic H1N1 probe and 2 μL RNA templates. The protocol of real-time RT-PCR for influenza A (H1N1) recommended by WHO used a concentration of 1000 nM each of novel SW H1 primers and 250 nM of SW H1 probe [18]. The WHO real-time RT-PCR protocol for H5N1 used a concentration of 800 nM each of H5 primers and 200 nM of H5 probe [19].

**Figure 1:**
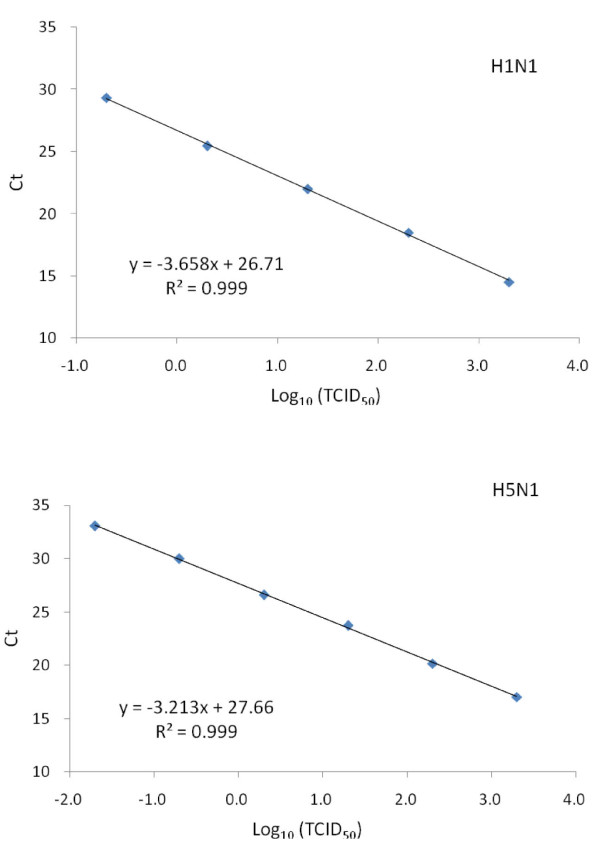
**Sensitivity and dynamic range of the duplex real-time RT-PCR assay for detection of H5N1 and pandemic H1N1 viral RNA**. Serial 10-fold dilutions of viral RNA standard (from 2 × 10^-2 ^to 2 × 10^4 ^TCID_50_) were plotted against the threshold cycle (Ct). A Minimum of 0.2 TCID_50 _H1N1 viruses or 0.02 TCID_50 _H5N1 viruses per reaction could be detected. The coefficient of determination (R^2^) and the equation of the regression curve (y) calculated.

The sensitivity of the single and duplex real-time RT-PCR for novel H1N1 and H5N1 virus was evaluated by 10-fold diluted virus RNA, respectively. The pandemic H1N1 influenza virus strain A/Beijing/501/2009(H1N1) (GenBank: GQ223408-GQ223415) and avian influenza virus H5N1 strain A/Beijing/01/2003 (GenBank: EF587274-EF587281) were cultured on MDCK cells and tested by the assay. The analysis of threshold cycles (Ct) signals as a function of log10 TCID_50 _titers of tested viruses showed a nearly linear decrease of Ct value with increased virus titer. The sensitivity of the single real-time RT-PCR assay was 0.2 TCID_50 _for both novel pandemic H1N1 and H5N1 virus. The detection threshold of the duplex real-time RT-PCR assay was 0.2 TCID_50 _for the pandemic H1N1 and 0.02 TCID_50 _for H5N1 virus. Compared with single real-time RT-PCR assay, the duplex real-time RT-PCR assay had the same sensitivity for pandemic H1N1 virus, and about 10-fold more sensitive for H5N1 virus (0.02 TCID_50_) (Figure 1). This was likely because the procedure of the duplex assay for H5N1 was optimized for Roche LightCycler 2.0 system, while the single real-time RT-PCR assay recommended by WHO was optimized for ABI instruments [18].

The specificity of the duplex real time RT-PCR assay was validated by using human genomes and a panel of respiratory tract viruses including human seasonal H3N2 influenza viruses, seasonal H1N1 virus, human respiratory syncytial virus A and B, human coronavirus 229E, human coronavirus OC43, influenza B virus, human parainfluenza virus and human adenovirus. All samples were tested negative (data not shown).

A total of 236 clinical throat swab specimens from suspected cases of novel pandemic H1N1 patients were collected. The samples were first detected by WHO real-time RT-PCR protocol for influenza A (H1N1). As the WHO protocol also included a set of primers for universal detection of type A influenza viruses, thus, only the 182 Influenza A positive samples confirmed by WHO protocol were further tested by the duplex system for comparison with the results of WHO protocol. Among the 182 influenza type A positive specimens, 124 specimens were positive for the pandemic H1N1 and none for the H5N1 tested by the duplex system. Duplex real-time RT-PCR assays showed 100% coincident results with the single real-time assays. Sequence analysis was also conducted with 20 samples selected randomly from the novel H1N1 positive specimens.

Since few clinical specimens was available currently in China, a mock clinical H5N1 specimen was extracted from the spleen of BALB/C mice infected with high pathogenic H5N1 influenza virus to validate the specificity of high pathogenic H5N1 virus. The duplex real-time RT-PCR assay showed strong positive result, which was confirmed by single real-time PCR and subsequent sequencing analyses. This demonstrated that the duplex real-time RT-PCR assay was a substitute method for the current single real-time RT-PCR method.

So far, the number of confirmed severe novel H1N1 cases has reached about seven hundred thousand in China and increased quickly. The lessons from 1918 and 1957 influenza suggested that the pandemic might return in a much more lethal form in the second wave [21]. Recently, the increasing geographic distribution of high pathogenic H5N1 as well as its ability to transfer to humans and to cause severe infection has raised serious concerns regarding the control measures of this virus. The duplex real-time RT-PCR assay for pandemic H1N1 and high pathogenic H5N1 would play an important role for control and prevention of pandemic caused by these viruses.

## Competing interests

The authors declare that they have no competing interests.

## Authors' contributions

XK and TJ: designed the study, did laboratory testing, analysed the test results, co-wrote and edited the manuscript. YL, FL, LH and GC took samples and did laboratory testing. QZ, CQ and YY organized the overall project and helped edit the manuscript. All authors have read and approved the final manuscript.
